# Experiences of well-being among female doctoral students in Sweden

**DOI:** 10.3402/qhw.v9.23059

**Published:** 2014-04-16

**Authors:** Manuela Schmidt, Timurs Umans

**Affiliations:** School of Health and Society, Kristianstad University, Kristianstad, Sweden

**Keywords:** Female, doctoral students, lifeworld, PhD students, phenomenological hermeneutics, well-being

## Abstract

The aim of this study was to explore how female PhD students experience and perceive their well-being. Focus groups were conducted with female PhD students employed at a Swedish university. The study was performed using a phenomenological hermeneutic approach based on the concept of the lifeworld, used as both a philosophical perspective and a methodology. Three main themes emerged from the analysis: *being true to oneself*, *being in the sphere of influence*, and *performing a balancing act*. By unfolding these themes, the study shows that perceptions and experiences of well-being in female PhD students are a multifaceted phenomenon and materialize through interaction of different aspects of “self” (agent) and “others” (structure). As well as illustrating these perceptions and experiences, the study also presents female PhD students’ conceptualization of their well-being, expressed in terms of a white-water rafting metaphor.

Women in academia still have problems finding their place in a world that over a long period of time has been strongly dominated by men, while achieving gender equality in academia has been an aim for several decades (Rees, 2001; Valian, 2004). Though today roughly as many females as males enrol in postgraduate programmes, it is still a world dominated by men because they hold positions with greater power, higher status, and higher salaries (Monroe, Ozyurt, Wrigley, & Alexander, 2008). In the United States, for example, although the percentage of women who enrol in graduate programmes has been above 50% for almost two decades, women account for only 44% of PhDs awarded, only 38% of the full-time faculty in all institutions of higher education, and only 14% of the tenured and tenure-track faculty in “top” departments (Monroe et al., 2008). In general, 80% of tenured professors are male (Monroe et al., 2008). In the United Kingdom, Australia, and New Zealand, the figures are very similar (Johnson, Lee, & Green, 2000; Nerad & Cerny, 1998; Ramsay, 2000; Thanacoody, Bartram, Barker, & Jacobs, 2006; White, 2003, 2004).

In Sweden, the situation in gender equality is by no means different. The number of female doctoral students increased from 23% in 1977 to 49% in 2010 (SCB, 2011a), dominating in research fields such as humanities, law, social science, and medicine, but in higher positions only 21% of tenured professors are women (SCB, 2011b). The fact that there is gender equality at the starting point of a career in a sense that the entrants into academia in Sweden are usually being judged by their competences and suitability for the open doctoral positions (Högskoleförordning, 1993:100) rather than being selected based on gender quotas, and gender inequality later on in higher academic ranks, indicates that something happens on the way, ranging from discrimination—expressed in terms of salary differences between men and women; resources allocation, which is still in many fields the male professors’ prerogative; and so on—to a conscious choice by women not to pursue a career in academia (Etzkowitz, Kemelgor, Neuschatz, Uzzi, & Alonzo, 1994; Menges & Exum, 1983).

Many authors have attempted to explain why women are more likely than men to leave the path to senior academic positions (e.g., Bellas & Toutkoushian, 1999; Dabney and Tai, 2013; Levinson, Kaufman, Clark, & Tolle, 1991; Menges and Exum, 1983; Quinn, 2011). Overall, the researchers agree that the positioning of women in academia and their experiences are being influenced by a number of exposures that originate from (1) the overall environment (e.g., societal sex role exceptions) (Menges & Exum, 1983), (2) more narrowly defined academic and work environments (e.g., the flexibility of the work schedule, an organizational culture supporting equality, the number of similar other women in the environment, and the availability of female role models at the top of the organization) (Kinman & Jones, 2008), as well as (3) individual and gender-specific factors (e.g., risk-taking capacity, stress tolerance, and family background) (Kundu & Rani, 2007). It is thus these structural, organizational, and individual factors that shed light on how inequality establishes and manifests itself in academia, yet it is not only the factors themselves but also experiences of these factors by women that might shed light on the inequality phenomenon in academia. One possibility to explore this matter further would be to turn to the other concepts these factors appear to shape and/or interact with, namely, well-being. We further argue that it is of particular importance to understand the well-being of female PhD students at the point in the female academic career where inequality appears to be less apparent than in further steps of the academic hierarchy, to shed light on the development of the academic career of women. In other words, we question the direct effect of various exposures on womens' academic development, instead posing that it is through understanding of experiences streaming from these exposures and manifested in the subjective gender-biased experiences of well-being (Kundu & Rani, 2007) that one can understand female career paths in academia.

While several authors have addressed the issues of well-being in PhD students (Haynes et al., 2012; Stubb, Pyhältö, & Lonka, 2011), most of the literature on the subject has been concentrated on isolated attributes rather than taking a more holistic perspective that takes into consideration a number of factors that shape well-being and interact with each other simultaneously (Moberg, 1979). The Literature Review section thus presents these findings and provides a rationale for applying a holistic experience-based perspective to the well-being of female PhD students.

## Literature review

Academic staff as an occupational group is worth investigating, especially in terms of their well-being, since it is they who ensure the quality of higher education institutions in both research and education. They represent the institution's key resource in the drive to reach and maintain the institutional goals (Machado, Soares, Brites, Ferreira, & Gouveia, 2011) that ultimately benefit the society through the creation and development of knowledge and innovation (Gillespie, Walsh, Winefield, Dua, & Stough, 2001).

Research on the well-being of academic staff (of which PhD students are a natural part) has shown that their well-being is usually shaped by self-perception and self-assessment (Beckman, Reed, Shanafelt, & West, 2010; Flaxman, Ménard, Bond, & Kinman, 2012; Puig-Ribera, Gilson, McKenna, & Brown, 2007), mental and physical health (Beckman et al., 2010; Flaxman et al., 2012; Hapuarachchi, Winefield, Blake-Mortimer, & Chalmers, 2003; Kinman & Jones, 2008; Puig-Ribera et al., 2007; Schindler et al., 2006; Vera, Salanova, & Martin, 2010), and supporting structures such as academic, social, and work environments (Beckman et al., 2010; Kinman & Jones, 2008; Puig-Ribera et al., 2007; Ronald, Mustafa, & Lisa, 2008; Schindler et al., 2006).

Doctoral students have been singled out as a special category among university staff for several reasons. Life as a doctoral student is often characterized by constant peer pressure, frequent evaluations, low status, high workload, paper deadlines, financial difficulties, pressure to publish, and active participation in the scholarly environment, including conferences (Kurtz-Costes, Helmke, & Ulku-Steiner, 2006; Tammy & Maysa, 2009). Often, entering PhD studentship is also associated with a sudden switch from a practical profession into the new or somewhat obscure world of academia (Holligan, 2005; Kurtz-Costes et al., 2006). While such issues could be generally attributed to PhD students (as a part of academic staff), it has been argued that they represent a specific occupational subcategory (Doyle & Hind, 1998) in which experiences of well-being might be attributed to a number of very specific, PhD studies–related contextual factors (Haynes et al., 2012).

Motivated by findings of recent research that attrition rates for women enrolled in PhD programmes are higher than for men (Castro, Garcia, & Castro, 2011; Mansfield, Welton, Lee, & Young, 2010; Marschke, Laursen, McCarl Nielsen, & Rankin, 2007), researchers have started to put particular emphasis on understanding what could be the reason behind this outcome. Our narrowing of the focus to the well-being of female PhD students was also influenced by studies showing that experiences of well-being differ between genders (Roothman, Kirsten, & Wissing, 2003).

Researchers active in this gender-oriented stream have found that female doctoral students have more difficulties in coping with their studies, triggered for instance by experiences with or lack of different support systems (Damrosch, 2000; Haynes et al., 2012; Juniper, Walsh, Richardson, & Morley, 2012; Kinman & Jones, 2008; Puig-Ribera et al., 2007; Pychyl & Little, 1998), difficulty navigating organizational culture and climate (Lovitts & Nelson, 2000; Rhode, 2003), or difficulties balancing work–family roles and financial and other obligations (Beckman et al., 2010; Haynes et al., 2012; Hubbard & Atkins, 1995; Juniper et al., 2012; Kinman & Jones, 2008; Moyer, Laovey, & Casey-Cannon, 1999; Pychyl & Little, 1998). Furthermore, unsatisfactory mentor–supervisor relationships (Ives & Rowley, 2005; Lee, 2008; Stubb et al., 2011) and lack of guidance are additional obstacles that might result in prolonged or noncompleted doctoral studies (Castro et al., 2011). Late entry to postgraduate study (Chesterman, 2001), part-time rather than full-time studies (White, 2003), feeling “marginalized” (Thanacoody et al., 2006), having responsibility for childcare (Jackson, 2008), and having a more complex life situation (Hill & McGregor, 1998) might deliver further reasons for the high attrition rates among female PhD students, according to the literature.

Some studies in the field (Doyle & Hind, 1998), however, argue that looking at isolated attributes of well-being might be futile, since it is the interrelations among those attributes of female PhD student life that could explain issues experienced by women during and after their doctoral studies in academia. Thus, while the aspects identified in this literature review offer an insight of what female doctoral students have to struggle with, further exploration of their *experiences* during their studies might shed light on the complexity of influences and interactions of the attributes of their well-being that were previously studied in isolation from each other. The purpose of this article is, therefore, to explore how female doctoral students experience and perceive their well-being.

## Method

The study adopted a phenomenological hermeneutic approach based on the concept of the lifeworld (Dahlberg, Dahlberg, & Nyström, 2008), which was employed as both a philosophical perspective and a methodology in this qualitative investigation. The only necessary requirement of lifeworld research is a fairly well-defined phenomenon as the focus of the study (Dahlberg et al., 2008). According to Heidegger, a phenomenon can be understood as an object, a matter, a “thing,” or a “part” of the world as it presents itself to, or as it is experienced by, a subject. Phenomenology is thus the science of the phenomena and, consequently, the science of the world and its inhabitants, with the “things of the experience” understood as the world of experience. Applying the hermeneutic approach, the author then attempts to understand the phenomenon by interpreting the participants’ experiences. This requires that the author (i.e., interpreter) must step into the world of the participants in order to fully understand their experiences. Schleiermacher calls this approach *Einfühlung*, meaning an attempt to reach an understanding of the participants’ minds and thus grasp their psychology. Putting aside the controversy between phenomenologists and hermeneutists, where the former accuse the latter of being speculative, and the hermeneutic researchers in turn argue that phenomenologists are being interpretative without knowing it and that it is impossible to describe since interpretation is the basic approach to the world, we have used a combination of both views in this study. With a phenomenological-hermeneutic approach, we attempt not only to describe human experience of the phenomenon of interest but also to interpret and understand it (Heidegger, Macquarrie, & Robinson, 1962).

Meeting the women from a lifeworld perspective means being able to see, understand, describe, and analyse parts of their world, for example their well-being as they perceive it during their PhD studies. The lifeworld perspective is formed by an interest in people's own stories (Dahlberg et al., 2008), so a hermeneutic approach was considered as highly suitable for this study. With openness as a foundation (Gadamer, 1997), women's experiences of well-being were interpreted to understand their meaning. A hermeneutic approach based on Gadamer's philosophy (Gadamer, 1997) regarding pre-understanding existential interpretations can be described as an attempt to understand how the women experience their life situations as doctoral students. From a hermeneutic perspective, the data that are compiled depend on interpretation and any relevant insight or understanding stemming from the authors’ background (Ödman, 2007).

### Participants

Female doctoral students employed at a university in Sweden were approached by one of the authors by either email or telephone and asked if they were interested in participating in a study about the well-being of female doctoral students. All students contacted were willing to participate; 12 women were chosen by purposive (or purposeful) sampling (i.e., typical representatives for this subpopulation were sought). In particular, maximum variation sampling was used to capture enrichments of and challenges to emerging conceptualizations (Polit & Beck, 2012). The selected participants were chosen on the basis of their variations in terms of age, ethnicity, field of study, and varied length of doctoral studies. Three focus groups were formed, mainly determined by convenience of time scheduling; this resulted in groups in which some of the doctoral students knew each other to some degree, and others did not know the people in their group.

Participants received more detailed information about the study and confirmation that participation would be confidential. There were no inclusion criteria other than being employed as a PhD student at a Swedish university for at least 2 months. The women's research fields varied from biology to business administration, health sciences, nursing, informatics, and public health. Their age varied from 31 to 50 years. Though it was not an inclusion criterion, all of them happened to be mothers of one, two, or three children and were in a stable relationship or married. Three of the doctoral students were about to hold their final defence of the dissertation; one had started her doctoral studies only 2 months prior to the data collection; the rest were spread in between these two stages. The majority of the women studied full-time (80 to 100); several studied part-time (50). The majority of the women were familiar with academia through working as an adjunct or research assistant prior to the interviews.

### Data collection

Data were collected through focus group interviews (Kitzinger, 1995; Krueger & Casey, 2009), a data collection method that meets the characteristics of human being and existence to which a lifeworld phenomenon such as well-being is associated (Dahlberg et al., 2008). In contrast to group interviews, focus groups pay particular attention to members’ interaction with one another, and the interactions thus form part of the research data (Kitzinger, 1994). The intention of using focus group interviews was to encourage those interactions between the participants as much as possible because when group dynamics work well, the participants act as co-researchers, taking the research into new and often unexpected directions (Kitzinger, 1994). The doctoral students were able to engage in interactions which were both complementary (such as common experiences) and argumentative (such as questioning and disagreeing with each other). This synergy, also referred to as the “group effect” by Carey (1994) and Carey and Smith (1994), offers valuable data, and it is this effect that makes focus groups more than the sum of individual interviews (Morgan, 1996). Yet we are aware of the criticism put forward by some researchers (e.g., Webb & Kevern, 2001) that find phenomenology to be incompatible with focus group interviews. However, in line with Bradbury-Jones, Sambrook, and Irvine (2009), we argue that experiences are seldom only individually based and usually are a product of interaction between the self and the environment. We further argue that only by elaborating on experiences in the group rather than in one-on-one interview settings are participants able to relate to each other's experiences and in this way produce a richer and more comprehensive account of their reality.

During the focus group interviews, each of which consisted of four participants, there was strong emphasis on encouraging conversation with each other. One of the authors, who participated as a moderator, concentrated on keeping the discussion flowing (Krueger & Casey, 2009). The interview guide (Kidd & Parshall, 2000; Krueger & Casey, 2009; Morrison-Beedy, Côté-Arsenault, & Feinstein, 2001) was prepared in advance and consisted of six questions that were used in all three focus groups. Firstly, the participants were asked to present themselves and to tell the others why they have chosen to become a PhD student. Further questions, all of an open nature, encouraged the participants to share with each other as well as discuss: the meaning of being a PhD student, experiences associated with being in this position, as well as what well-being means to them. These questions aimed at allowing the women to discuss their experiences of being a PhD student, to relate to each other, and to bring up their individual experiences of well-being. The topic guide for the follow-up group interviews consisted of five predefined questions. Questions here have been developed based on the discussions that arose during the first focus group interviews but were not sufficiently discussed due to the lack of time. For example, participants had been asked to discuss and reflect on their experiences in specific contexts (e.g., home, work, and conferences). During the interviews, the participants were given the freedom to bring up anything they wished, and this influenced the direction of the discussion; this design classified the focus groups as relatively semistructured interviews and suitable for this study since openness, as taught in hermeneutics, still requires order and structure. An open and rather flexible interview style was adopted when leading the focus groups; this also is in line with the hermeneutical approach. One might criticize the fact that instead of letting the participants lead the discussion in any direction possible, we set loose boundaries of the discussion by using some predefined questions. While aware of this shortcoming, it was a conscious choice to somewhat guide the discussion. This choice was made since one of the authors of this article has had previous experiences in conducting focus group interviews with PhD students, and often the discussion has led to an exchange of experiences connected to tasks performed within the PhD position. By avoiding task-related discussions in the group, we have somewhat reduced the validity of the results, yet we have increased the possibility of the groups to discuss experiences of well-being, which thus matched the aim of this study. However, it is important to stress that the questions were not imposed on the participants and have been asked only if silence prevailed in the group for longer than what is normally deemed comfortable. Two of the three focus groups^1^ were interviewed twice in order to provide the opportunity to ask follow-up questions and gather information in depth.

In four out of the five (i.e., three initial focus group interviews and two follow-up focus group interviews) instances when focus group interviews were performed, an observer was present to concentrate on significant nonverbal communication, emotions, interactions among the participants, and dynamics within the group. The observers were female and either a lecturer or another doctoral student. After each interview, the moderator and observer discussed their impressions.

The focus group interviews took place during the spring of 2012 at the university where the doctoral students were employed. The interviews were held in a room that was “neutral” and quiet; beverages and snacks were served in order to put participants at ease. The discussions lasted approximately 1 h 30 min and were audio-recorded before being transcribed verbatim. All identifiable names were removed from the transcripts.

In addition to the focus groups, some demographics and other information about the PhD studies were collected from each participant. A short questionnaire with items about the participant's age, civil status, number of children, date and place of enrolment, degree of employment (as a percentage), and type of employment before enrolment was sent via email before the interview took place as well as handed out after the interview.

Further, a research diary was kept by one of the authors to record not only her pre-understanding of the phenomenon under study, but also all ideas, thoughts, and questions raised during the study; these were noted immediately throughout the research process, particularly during and after the focus group discussions. The diary was an important element during the analysis.

## Analysis

Data were analysed based on Dahlberg's principles of the lifeworld (Dahlberg et al., 2008). The lifeworld can be seen as a base of phenomenological philosophy and existential hermeneutics. Well-being is a lifeworld phenomenon (Dahlberg et al., 2008). In this study, we describe the lifeworld of the female doctoral students so as to gain a better understanding of the aspects that are important for their well-being.

The analysis was divided into three phases. The first step was to read the text as a whole—the initial reading (Dahlberg et al., 2008). This implied that all the transcribed interviews were read in order to obtain a sense of the whole. Though interpretation was not included in this phase, the authors entered into a dialogue with the text. Having gained a preliminary understanding of the data, a new dialogue with the text began. The second step was to divide the whole text into meaning units (Dahlberg et al., 2008) which then were condensed; that is, the essential meaning was expressed in abstract subthemes which then were assembled into themes. Lindseth and Norberg describe this process as a structural analysis; the process is repeated until one feels that the initial understanding is validated through the structural analysis (Lindseth & Norberg, 2004). In the third step, all parts were put together in a new way to create a new whole, and thus new understanding (Dahlberg et al., 2008).

The authors of this article have both performed the three steps of the analysis independently from each other, after which the results of the analysis were compared and discussed, which led to the repetition of the three steps now with both authors being aware of the each other's analysis as well as pre-understandings (embedded in the differences of academic status and experiences, as well as cultural and gender differences among the authors) that the authors have revealed and discussed with each other prior to the second wave of the analysis. After that, the authors again compared the results of their analysis and possible and smaller deviations, after which the arrival to the common understanding and interpretation was established.

### Ethical considerations

The study has adhered to the basic principles for research given in the Helsinki Declaration (World Medical Association, 2008). We refer to the applicable paragraphs of Helsinki Declaration in prentices while describing procedures performed when conducting this study. The participants were informed about the study and that participation was voluntary (B.22). Prior to the focus group interviews, the participants received an email briefly describing the project and repeating the topic to be discussed (B.24). Furthermore, it provided one of the author's contact details and stated that the discussions would be audio-recorded for later transcription (B.24). A consent agreement was signed by each participant that allowed use of the material for the purpose of this study (B.24); it reassured the participants that their identities would not be revealed (B.11; B.23). Since the discussion was entirely based on mutual understanding and agreement, participants had the choice to leave at any time and share only information they felt comfortable with (B.24). During the interview, both the observer and moderator tried to show sensitivity and understanding to the participants and the topic at hand (B.11). The moderator's role has been taken by a researcher with appropriate scientific training and qualifications (B.16). There was no dependency relationship between the researchers performing the study and the interviewees (B.26). Data were stored safely and were available only to the authors of this paper. Ethical approval was not sought for the study since data collected was of non-biomedical nature (SFS 2003:460, 2003; SFS 2008:192, 2008) yet the researchers have adhered to and considered the ethical, legal and regulatory norms and standards for research involving human subjects in Sweden and internationally by adhering to the SFS standards mentioned above (A.10; B.12). Finally all the participants of the study have been provided with the draft including the theoretical frame (B.12) and the initial results of the study (C.33).

## Results

Here, we present three themes which were identified in the structural analysis and illuminate the lifeworld of female PhD students and how they experienced well-being. This is followed by a comprehensive understanding that completes the analysis.

### Being true to oneself

Female PhD students’ well-being is coloured by their overall approach to life and their relationship with the self, that is, self-perception, personal expectations, acknowledgement of one's limitations in terms of ability, and so on. If they strive for new knowledge, understanding is of existential importance: the person defines herself through the eyes of a researcher, and the PhD studies are given a very high priority. For participants of this study, however, the PhD studies were seen as a part of everyday life (Table I).

**Table I:** Theme “Being true to oneself”: Subthemes and examples.

Subthemes	Examples of meaning units
Knowing oneself	I have never aimed at being there, far ahead [PhD], but … it's only circumstances that have … made that I moved on.
	I'm not looking for any titles.
	I live in the present, I'm here and now.
Being able to prioritize	My job is no more important than my family.
	The rest of the time, we have to do everything else that needs to be done in life.
	They [children] have a hard time understanding that the studies have a higher priority than family—it's very difficult for them.
Being the chosen one	I am chosen—self-affirmation … a bit of egoism in the whole thing, from my side.
	It's not just about the children—this is about myself.

Rather than seeing PhD studies as an end to the means, generally the students saw PhD studies as a process through which they set out on the road of lifelong learning. In general, respondents felt a positive development of their well-being was due to adopting a process- rather than goal-oriented approach in relationship to their PhD studies.

Almost all students stated they were unwilling to compromise their private life for the sake of succeeding in their studies. Yet many of them admitted that at times when the workload became heavier and in stressful situations (typically before submitting a paper, middle or final seminars, teaching in combination with research, presentations, or participation in conferences), other parts of their lives suffered and led to feelings of guilt, frustration, and bad conscience. In a few cases, it required a personal crisis before they realized that other parts in their life besides study had to be prioritized for their own well-being.

The participants became PhD students for different reasons, but few described their choice as a calling, a strong desire, or a long-term plan they worked towards; rather, they considered it as a combination of different factors like curiosity, coincidence, or seeking a challenge. Most students defined being a PhD student as a journey, rather than a destination, and the majority of them already were familiar with or working in academia, for example as an adjunct, research assistant, or student. Most of them were persuaded by other individuals (like potential supervisor(s), managers, or colleagues) to become a PhD student; only a few of them emphasized the desire for new knowledge or wanting to become a researcher as the major force. Depending on the reasons and the motives for becoming a PhD student—whether they were persuaded or the choice was individually made—determined whether they viewed the studies as a challenge, privilege, burden, or opportunity; as a normal working job with limited working hours; or as a lifestyle. The way into the PhD studies could also determine the level of ambition and to a great extent how flexible the students wanted or chose it to be, for instance in terms of working schedule or workload as well as the willingness to conform to expectations from the academic community or supervisors. Yet, while most participants stressed the process of PhD studies as important, some of them subtly made the point that process without a goal might be meaningless. Thus, while not having a clear goal orientation, participants indicated that interaction between process and goal was an important aspect of their being.

### Being in the sphere of influence

Though the women were aware that other research groups or universities cultivated a much more competitive working climate and that there were students, mainly men, who had a different approach to their studies and working time, they tried not to conform to this approach and appreciated a friendlier and healthier work environment that was not determined by competitive thinking (Table II).

**Table II:** Theme “Being in the sphere of influence”: Subthemes and examples.

Subthemes	Examples of meaning units
Being part of scholarly community	It's a tough business.
	You're at the bottom of the scale here, it's not that remarkable. Where I'm enrolled, there is tremendous competition.
Being in a man's world	There is a difference between how male and female doctoral students are treated.
	Men take more space than women and I'm thinking: “Now we are here in tertiary education and it's exactly the same—how is that possible?”
Playing by new rules	We do as we want and feel like without following—if they exist—those norms and unwritten rules.
	But then, it's good if someone dares to go against the grain too.
	I think it is important that we set boundaries.
Being understood by peers	We understand each other and we have each other at meetings.
	[It's good] to have someone who is in the same situation …
	There is no one else that understands you as well as another doctoral student, I feel.
	They know what … what position … how vulnerable one is.
Being mentored by supervisor	The supervisor is everything.
	What is negative is how tied one is to this supervisor, it's disgusting.
	One does live in a dependent situation.
	I think I have been much influenced by my supervisor: he somehow put up the rules of the game and I had to follow them.
Being supported by family	They are a bit proud as well.
	It's all about having an understanding partner.
	They probably feel that they have to sacrifice a lot, and I have a bad conscience because of that.
	Support from home is extremely important.

Furthermore, the participants also noticed that in certain research fields, PhD students chose to embark on this path at a relatively young age, with career growth and/or financial gain being the primary reasons for this endeavour. It was noticeable that the women distanced themselves from these goals, claiming that with age the importance of these goals subsided and other goals such as well-being became of higher concern and priority.

The students were very aware of peer pressure but chose not to engage in this in order to feel better and less stressed. They conformed to a certain extent at the beginning of their studies because they did not know the rules and norms imposed by the scholarly community, their supervisors, or their research groups and did not know what worked best for them; but as soon as they realized that certain things made them feel worse or when they came to a life crisis, they took hold of the issues that they did not like, took the initiative, and tried to change the situation for the better. They also saw their studies as a long-term commitment; that is, they needed to find a way that would allow them to get to the end of the programme and achieve well-being throughout the whole process.

The need for stability and structure for the women was a reoccurring theme throughout the interviews. Female PhD students went through many ups and downs in a short period of time that could be described as a mental roller-coaster ride; stability in other aspects of the studies—and other domains of life—was an essential requirement that added to their level of well-being. Stability and structure could be provided in different ways: for example, through a clear study plan, clear instructions about procedures at the institution, clear course plans, clear and satisfying working conditions, an understanding partner in a well-functioning relationship, and/or a supportive supervisor and colleagues. Being surrounded by other doctoral students was valued very highly. Exchanges of information, feelings, help, and guidance and feeling understood by someone who was in the same position were described as immensely important for their well-being and for succeeding in their studies. Another very influential factor for most students was the role played by the supervisor, to the point that they described themselves as being fully dependent on that person. The mentoring style and availability of the supervisor were crucial for successful supervision.

Interpersonal relationships were a very important cornerstone of students’ well-being. Since all of them were in a relationship and were mothers, the family situation played a significant role in all the participants’ lives. In many cases, when committing to PhD studies, financial sacrifices had to be made. Since most women still found themselves in a situation where loans for housing and expenses for children had to be covered before they started their study programme, their partners had to be able to cover these costs so they could maintain a similar lifestyle, and this meant some women found themselves to be financially dependent on their partners. So it was important for them to feel supported by their partner in their choice of career; otherwise, it could result in a stressful situation that could block the creative process at work and have negative impact privately. Keeping in mind the lengthy duration of PhD studies, financial issues could easily turn into a potential continuing focus of concern.

### Performing the balancing act

This theme (Table III) is characterized by many clashes. Being a PhD student was perceived as a very positive experience. Regardless of what the motives were for becoming a PhD student, they engaged in the subject because most of them were genuinely interested in their research field, not for career opportunities but rather to grow as a person or contribute to society.

**Table III:** Theme “Performing the balancing act”: Subthemes and examples.

Subthemes	Examples of meaning units
Being in or out of control	Right now I'm in such a period of frustration.
	Sometimes I could feel totally frustrated because I have been so free, I haven't got any control or guidance at all. It's such a time pressure the entire time … I'm breaking down.
	I like working alone.
	I enjoy being alone.
Living up to high expectations	Yes, it feels great; at the same time I'm under extreme pressure.
	I am feeling an internal stress now.
	Why do I get so stressed if someone says something?
	And then suddenly your confidence level hits the bottom, and it feels really horrible, shameful to even talk about it; it's only when it's over that you can talk about how it was.
Living a dual life	Being a PhD student is a bit more special than other jobs, because you always have it with you.
	[I] try to find a balance in life; I also plan my private life around my studies.
	… And then I realized, no, the job isn't everything.
	Because it's quite special being a doctoral student. And if it doesn't work at home, it's not easy to study, I think.
Being a working student	It's a job for me, it's definitely a job for me.
	It's a job, it's 40 h a week, just in order to be able to make it to the end and to be creative.
	It becomes a lifestyle … kind of, and you have it with you the entire time.
	No, I actually don't see it as a job, it just is. It's around the clock.
	But it's a lot like … that this is an education and you … yes, you are expected to work more hours than you have.
Being superwoman	It's actually me who is responsible for almost everything at home with children and family; it makes it harder to prioritize.
	I cried, that was the only thing I could do to get out the frustration, I felt like a horrible mother.

The female PhD students needed to like the topic of interest and working place in order to make it through all the way. Most of the participants perceived their studies as positive, stimulating, and challenging for their personal development. Hardly any plans were expressed about how they saw their journey continuing after graduation—which was not surprising, since they were not goal oriented but rather concentrated on maintaining well-being all the way to graduation and then letting circumstances decide how the journey would continue. Most of them lived “in the moment” and tried to take care of themselves and their families and to make sure their vocation added to their well-being and self-fulfilment. Though they described their studies as a very positive experience and a means toward self-fulfilment, the conversations in the interviews were often dominated by discussions about stress, pressure, problems of different kinds, and issues about combining their studies with their private lives.

Even though time was mentioned often and turned out to be one of the biggest stressors for study participants, it was also perceived by many as something very positive in terms of flexibility in working hours. Participants also mentioned how well it suited them to work alone and how this added to their independence and flexibility, but at the same time they needed much support from others and enriched channels for exchange of information. The need for structure and stability put students in a tension field of being independent in many ways, but too much independence could result in counterproductivity, dissatisfaction, and loneliness.

Expectations were very high on many levels. Not only did the women have to face their own demands and hopes, but their surroundings imposed high expectations on them as well. First, the scholarly community in general and the main supervisor in particular had specific ideas, wishes, demands, and expectations that were laid on the student. Then, the family situation could put her under pressure and cause stress in terms of expectations about such things as deadlines for completion of studies, finances, and time schedules—all potentially leading to an imbalance in their life.

As a female PhD student, one had to attend to many different roles not only at work (e.g., being a student, teacher, colleague, and employee) but also at home with the family (being a mother, wife or partner, daughter, and sister) and in private settings (being a friend; being a member in a political club or a hobby group). Sometimes, clashes could occur; one of the hardest undertakings in the whole process of being a female PhD student was to maintain a healthy balance in life, that is, finding time for family and children, engaging in the relationship with the spouse or partner, attending to studies and related work such as teaching, and not losing the self in the whole process. Relationships with friends, parents, or other relatives; hobbies; and so on needed to be nurtured as well in order to keep a healthy balance. But it was just this struggle of dividing one's time between all the tasks, using time efficiently, and juggling the various roles that often resulted in time pressure. This in turn could lead to stress in one domain of life, such as parenting, studying, or teaching, which might then easily take over other aspects of life—all of which might intensify the woman's well-being in both negative and positive ways. Feelings of guilt and shame were often the results of this chain reaction.

There was somewhat of a conflict in the participants’ arguments. On one side, they were fully aware of their rights and wanted to be mothers, wanted to be with their family and attend to their self-concepts, and tried to see their PhD studies as an “ordinary job”—as expressed by many of the participants; but, on the other hand, many of them prioritized their studies when necessary in certain situations, knowing well that it would affect their well-being and their private life in a negative way.

### Comprehensive understanding

The well-being of female PhD students appears to be shaped by a number of factors to which they attribute different levels of importance. These are external factors such as the significant others (to whom doctoral students compare themselves, or from whom they seek and receive feedback) and study- and work-related conditions (workload and associated feelings). Then, different individual attributes of “self” appear to be reflected in their well-being: factors such as self-perception and self-awareness, among other factors. Finally, in this study, the interaction between external and individual factors was shown to comprise the experiences of well-being in the women. It is the balancing act they perform that appears to define this interaction and to thus epitomize their well-being. The combination of positive and negative experiences embedded within these aspects and their interaction define their well-being. Either in combination or in sequence, these positive and negative experiences can be represented by a white-water rafting metaphor. Issues such as the “ups and downs” of the ride, the speed and direction of movement, the co-paddlers, the comfort of the journey, obstacles on the way, and the positions and roles of the co-paddlers (e.g., other PhD students) may represent these students’ experiences and define the attributes of their well-being. Picking up on this metaphor, the “Discussion” section that follows will elaborate further on these attributes and position them in a wider theoretical context.

## Discussion

The aim of this study was to explore how female doctoral students experience and perceive their well-being, attempting to answer the question by analysing well-being as a lifeworld phenomenon. The main findings were that female PhD students experience their well-being as being torn between their own values, perceptions, and priorities, on one hand, and, on the other, the external sources by which they are influenced and/or on which they depend, as well as the fact that they have to fulfil multiple roles simultaneously within an overall sustained life balance.

As outlined in the “Results” section, conceptualization of well-being in female PhD students can be expressed in terms of a white-water rafting metaphor. The ride on the fast-flowing river represents the way from point A (enrolment) to point B (dissertation). The properties of the river, its angles and ups and downs, shape the experiences of the ride (PhD programme). The rafting boat represents the environment (usually the workplace or study place) and defines the level of comfort of the ride as well as experiences during the ride. The co-paddlers (peers, supervisors, family, and others) sharing the boat also shape the experiences of the ride and the movement of the boat (via supportive roles and common interests in moving forward). Friction between these people may also affect the movement of the boat (e.g., working with or against each other and affecting the balance). Moreover, there are different types of interactions between the co-paddlers which define the experiences of the ride (these interactions can be defined in terms of formality and informality, quality, rules, the frequency of communication, task and interpersonal conflicts, and the social integration of people with each other and with the PhD student).

The “self” and the role(s) one undertakes during the ride also shape one's experiences because, depending on the attributes of the “self,” various events in the ride from A to B are perceived in different ways. For example, how one experiences the speed of the ride, the comfort, or the braking and other movements forward or backward is shaped by one's own perceptions that also might change over time. Moreover, during the ride (PhD studies), the female PhD student can choose one of several roles: being (or, as our data show, rather not being) the leading paddler in actively directing and thus influencing the path (or, as our data have shown, trying not to get into that position), the co-paddler (letting the significant others and events determine the direction and speed of movement, while concentrating on other paddlers’ needs), or even the passive onlooker (being able to mentally [rather than physically] rise above the situation to observe and reflect).

Viewed from the white-water rafting metaphor, the experiences of well-being are multidimensional; different aspects and changes in them emerge as important attributes of well-being. One the one hand, well-being appears to be shaped by the PhD student herself and the role she adopts; on the other hand, well-being is represented by various types of external factors, such as influential others and societal pressure to maintain balance and not deviate from the course chosen.

It appears that female PhD students’ well-being finds itself cramped in the interaction between self and structural forces, which resonates well with Giddens’ structuration theory (1984). Claiming that a social phenomenon (in this article, the experience of well-being) emerges in the interaction between the agent (the female PhD student) and the structure (the societal structures within which the student is positioned), Giddens rejects the notion of independence of agency and structure that is dominant in social science research (Jones & Karsten, 2008). While some researchers have claimed that Giddens’ structuration theory is irrelevant for empirical research (Gregson, 1989), a number of authors have successfully been able to apply a set of ideas of the theory to empirical studies (e.g., DeSanctis & Poole, 1994; Orlikowski, 1992). While we have found no studies of well-being connected to structuration theory, gender-oriented studies have actively explored the theory in trying to understand gender as a social structure (Risman, 2004). Gender-oriented studies have combined structuration theory (Giddens, 1984) with the structural theory of action (Burt, 1982), the latter arguing that actors compare themselves and their options to those in structurally similar positions (Risman, 2004). Drawing on the interaction between structure and agent, gender researchers have thus theorized that women will seek to maximize their well-being by comparing themselves to men and other women (other agents) as well as taking environment into account. According to Risman (2004), experiences of well-being in women will arise as an outcome of the social-structural constraints.

The present study illuminates how well-being in female PhD students is experienced through interaction of the agent and structure and by doing so reaches an understanding of the conceptualization of this social phenomenon, representing the theoretical contribution of this article.

Apart from contributing to illumination of the interaction of agent and structure in the conceptualization of well-being, the analysis of the empirical material has shown that interaction of the agent and the structure creates tensions in the experiences. These tensions are expressed through the contradictions of well-being experiences, ranging from love to hate, from joy to sorrow, from excitement to depression, and being dependent on the particular stages of the journey, co-paddlers, and one's own feelings, among others. These issues were reflected in the themes that emerged in the analysis of the empirical data. The themes (*being true to oneself*, *being in the sphere of influence*, and *performing a balancing act*) have been partly touched upon in previous research (e.g., Ives & Rowley, 2005; Lee, 2008; Stubb et al., 2011), yet past research has often been one-sided in that it has only explored the isolated antecedents of well-being without paying attention to the antecedents’ interaction. Empirical findings of our present study, however, show that in order to understand the well-being of female PhD students, one needs to take a holistic approach and investigate how attributes interact with each other. By presenting the interaction of the antecedents and by allowing female voices to be heard, this article's empirical contribution is expressed in terms of exploration of interaction and even conflict among the various attributes of well-being in female PhD students.

Practical implications of this article are expressed in terms of illumination of various determinants of female PhD students’ well-being that can form a base for the development of educational policy in institutions’ PhD studies programmes. Moreover, the findings of this article might be used as a guide for PhD students to help them understand their experiences and in turn allow them to be more aware of the obstacles and facilitators that might be appearing on their way up the academic hierarchy.

## Limitations and future research

This study is not without limitations. First, the limited number of observations is a potential threat to the study's trustworthiness, yet different methodological remedies have been taken to ensure the critical evaluation and re-evaluation of the outcomes. Second, performing a study in a single context could be yet another threat to trustworthiness, yet the aim of the study was not to make a statistical generalization, but rather to be able to generalize analytically (Yin, 1993). Third, one cannot discount potential threats to trustworthiness of this qualitative study (Guba & Lincoln, 1994; Lincoln & Guba, 1985). Yet the study has been rigorous in securing credibility—by providing an authentic account of the participants’ experiences and being transparent about procedures of the data collection; assuring dependability—by carefully crafting the topics of discussion for the focus group interviews; confirmability—by attempting to let the respondents’ voices rise above that of the interpreter; transferability—by being open in describing the participants and their backgrounds; and authenticity—by presenting samples of citations of the respondents for each subtheme. Since all participants were mothers and lived in stable relationships, another limitation of the data could be seen in this homogeneous group in terms of transferability, but since qualitative research focusses its attention on depth rather than breadth (Ambert, Adler, Adler, & Detzner, 1995; Thomas & Magilvy, 2011; Whittemore, Chase, & Mandle, 2001), the material collected aimed at exactly that.

Further studies are needed to investigate attributes of well-being in gender-mixed focus groups to explore the differences of experiences between men and women. In addition, these studies might aim at performing quantitative investigations of specific attributes of well-being and their impact on PhD student effectiveness, efficiency, and productivity, among others.

## Note

1 The focus group interviews were performed at the end of the spring semester, and unfortunately the participants of the third focus group could not be easily reached and organized for the follow-up discussion due to the summer vacation.
